# Precision targeting of type 2 inflammation in COPD: identifying candidates for dupilumab therapy

**DOI:** 10.3389/fphar.2026.1971309

**Published:** 2026-10-12

**Authors:** Kezhuo Zhong, Kaiying Lv, Qun Liang

**Affiliations:** 1 Heilongjiang University of Chinese Medicine, Harbin, China; 2 The First Affiliated Hospital of Heilongjiang University of Chinese Medicine, Harbin, China

**Keywords:** biologic therapy, biomarkers, chronic obstructive pulmonary disease, dupilumab, eosinophils, type 2 inflammation

## Abstract

Type 2 (T2) inflammation represents a clinically important and therapeutically tractable endotype in 20%–40% of patients with chronic obstructive pulmonary disease (COPD). This subgroup is characterised by elevated blood eosinophil counts, increased fractional exhaled nitric oxide (FeNO), and activation of the interleukin (IL)-4/IL-13 pathway, and is associated with higher exacerbation risk and greater corticosteroid responsiveness. Dupilumab, a fully human monoclonal antibody that blocks the shared IL-4 receptor α subunit, became the first biologic approved for COPD following the replicate phase 3 BOREAS and NOTUS trials, which demonstrated approximately 30%–34% reductions in moderate or severe exacerbations in eosinophil-enriched populations receiving triple inhaled therapy. Pooled analyses have reinforced these findings, while post-hoc biomarker studies show that higher baseline eosinophil counts and FeNO independently predict greater treatment response. Dual IL-4/IL-13 blockade also reduces circulating IgE, eotaxin-3 and pulmonary and activation-regulated chemokine, providing mechanistic evidence of pathway modulation beyond eosinophil depletion. Patient selection should prioritise individuals who meet the pivotal trial criteria, confirmed COPD, frequent exacerbations despite optimised triple therapy, blood eosinophils ≥300 cells/µL, and chronic bronchitis features, while recognising that lower eosinophil thresholds and additional T2 markers remain incompletely validated. Real-world evidence remains sparse and limited by small sample sizes and observational designs. Realising the full potential of T2-targeted therapy will require pragmatic trials with broader inclusion criteria, prospective validation of multi-marker strategies, longer-term outcome data, and head-to-head comparisons with other biologics.

## Introduction

Chronic obstructive pulmonary disease (COPD) is a common, preventable, and treatable respiratory condition characterized by persistent respiratory symptoms and airflow limitation that is not fully reversible. The diagnosis rests on post-bronchodilator spirometry showing a forced expiratory volume in 1 s to forced vital capacity ratio (FEV_1_/FVC) of less than 0.70(1). Globally, COPD is a leading cause of morbidity and mortality, affecting approximately 10% of the adult population over 40 years of age in most general-population surveys, with substantial regional variation and an increasing burden in low- and middle-income countries (Bhatt et al., 2023; Rabe et al., 2023). The disease results from gene–environment interactions over the life course, with tobacco smoking being the predominant risk factor in high-income countries, while biomass fuel exposure, occupational dusts, and childhood respiratory infections contribute importantly in other settings. Major comorbidities, including cardiovascular disease, metabolic syndrome, osteoporosis, depression, and lung cancer, frequently coexist and influence prognosis independently of airflow limitation. The Global Initiative for Chronic Obstructive Lung Disease (GOLD) provides an internationally accepted framework for diagnosis, assessment, and management, stratifying patients by symptom burden, exacerbation risk, and spirometric grade to guide initial pharmacological therapy.

Asthma is defined by variable and often reversible airflow limitation, whereas COPD is characterized by persistent airflow limitation that is not fully reversible; a subset of patients has overlapping features. Although COPD has traditionally been viewed as a neutrophil-driven disorder, 20%–40% of patients exhibit concurrent type 2 inflammation, marked by elevated blood eosinophils, increased fractional exhaled nitric oxide, and activation of IL-4/IL-13 signalling pathways (Rabe et al., 2023; High et al., 2024; Sandelowsky et al., 2025; Vanetti et al., 2025). This T2-high endotype identifies a biologically distinct subgroup with a higher exacerbation risk, greater symptom burden, and enhanced responsiveness to inhaled corticosteroids, thereby providing a compelling rationale for targeted biologic therapy (Maniscalco et al., 2025). Dupilumab, a fully human monoclonal antibody that binds the IL-4 receptor α subunit and blocks both IL-4 and IL-13 signalling, became the first biologic approved for COPD after the landmark BOREAS and NOTUS phase 3 trials demonstrated approximately 30%–34% reductions in moderate or severe exacerbations among eosinophil-enriched populations (Bhatt et al., 2023; Bhatt et al., 2024). Pooled analyses and network meta-analyses have subsequently reinforced these efficacy findings (Bhatt et al., 2025a; Li et al., 2025; Pitre et al., 2025). Regulatory approvals in Europe and the United States in 2024, together with evolving guideline recommendations, including the 2026 GOLD report^1^, which now includes a section on biologic therapy for T2-high COPD and incorporates dupilumab into the exacerbation follow-up pathway, the NICE guideline (National Institute for Health and Care Excellence, 2026) TA1142 (2026) recommending dupilumab for uncontrolled COPD with blood eosinophils ≥300 cells/µL, and the Spanish GesEPOC 2025 update (Miravitlles et al., 2025), which has incorporated biologic therapy for patients with eosinophilic COPD and mucus hypersecretion as a treatable trait, have positioned dupilumab as a precision-medicine option for a carefully defined subset of patients with COPD and T2 inflammation. Mepolizumab, an anti-IL-5 monoclonal antibody, has also shown benefit in the MATINEE trial, whereas results for benralizumab and tezepelumab remain mixed or inconclusive (Celli et al., 2025; Sciurba et al., 2025). This perspective synthesizes current evidence on the T2 inflammatory phenotype in COPD, patient selection for dupilumab, biomarker stratification, and real-world implications.

## The type 2 inflammation phenotype in COPD

Type 2 inflammation is a distinct immunological effector response, originally characterized in the context of helminth infection and allergic diseases, that is now recognized to operate in multiple tissue compartments—including skin (atopic dermatitis), nasal mucosa (chronic rhinosinusitis with nasal polyps), oesophagus (eosinophilic oesophagitis), and the lower airways (asthma and a subset of COPD). The Type 2 response is orchestrated by T-helper 2 (Th2) cells, group 2 innate lymphoid cells (ILC2s), and, to a variable extent, mast cells and basophils, which produce the signature cytokines interleukin (IL)-4, IL-5, and IL-13 (Scott et al., 2023). These cytokines drive eosinophil differentiation, recruitment, and survival (IL-5); immunoglobulin E (IgE) class-switching and mast cell sensitization (IL-4 and IL-13); and goblet cell metaplasia, mucus hypersecretion, and airway smooth muscle remodelling (IL-13) (Barnes, 2016). By contrast, Type 1 inflammation is dominated by interferon-γ and tumour necrosis factor-α, orchestrating responses against intracellular pathogens and characterized by macrophage activation and cytotoxic T-cell responses; Type 3 inflammation features IL-17 and IL-22, driving neutrophil recruitment and responses to extracellular bacteria and fungi. These three major inflammatory axes are not mutually exclusive and may coexist in complex diseases, but the predominance of one over the others has major implications for biomarker selection and therapeutic targeting (Pesic et al., 2025). In COPD, while the classical description has emphasized Type 1 and Type 3 neutrophilic inflammation, 20%–40% of patients exhibit a T2-high signature that provides the biological rationale for dupilumab and other T2-targeted biologics.

The T2-high endotype in COPD is not defined by a single biomarker but rather by a constellation of clinical and molecular features. Blood eosinophil count remains the most widely used and accessible biomarker, with a threshold of ≥300 cells/µL adopted in the pivotal dupilumab trials; however, lower thresholds (≥150 cells/µL) may also identify patients with clinically meaningful T2 inflammation (Bhatt et al., 2023; Rabe et al., 2023; Vanetti et al., 2025). Fractional exhaled nitric oxide, total immunoglobulin E, eotaxin-3, and pulmonary and activation-regulated chemokine (PARC) provide complementary information about IL-4/IL-13 pathway activation, although their routine clinical utility is still being defined (Christenson et al., 2025a). Mechanistically, IL-4 and IL-13 drive key pathological processes in the airway, including eosinophil recruitment, goblet cell metaplasia, mucus hypersecretion, and airway remodelling (Scott et al., 2023; Song et al., 2026). Importantly, these structural effects are not fully captured by eosinophil counts alone, which may explain why dual IL-4/IL-13 blockade has broader biological effects than selective eosinophil depletion (Scott et al., 2023; Pescaru et al., 2026).

Clinically, patients with T2-high COPD frequently exhibit a chronic bronchitis phenotype with persistent productive cough, recurrent exacerbations, and a partial response to inhaled corticosteroids (Rabe et al., 2023; Maniscalco et al., 2025). Table 1 summarizes the key biomarker, clinical, pathobiological, and prevalence domains that define the T2-high phenotype in COPD and their clinical relevance. The co-occurrence of these features strengthens the likelihood of a T2-driven process and provides a practical basis for phenotypic recognition in routine practice. Nevertheless, blood eosinophil levels correlate imperfectly with lung tissue eosinophilia and can fluctuate over time, indicating that a single measurement should be interpreted in the context of the overall clinical picture (Criner et al., 2026; Staples et al., 2025). A systematic search of ClinicalTrials.gov and related registries (as of September 2026) confirmed that no dedicated phase 1 or phase 2 trials of dupilumab were conducted specifically in COPD. The clinical development programme advanced directly to the two pivotal phase 3 trials (BOREAS, NCT03930732; NOTUS, NCT04456673), leveraging extensive prior safety and pharmacokinetic data from approved indications in other type 2 inflammatory diseases. This approach is consistent with the established safety profile of the agent across multiple T2-driven conditions. Post-approval, several phase 4 and observational studies are ongoing or planned, including AEOLUS (NCT07053423; evaluating effects on airway inflammation, mucus plugging and remodelling by imaging), a post-hospitalisation exacerbation prevention trial (NCT07587658), and real-world registries such as AURORA (NCT07380711) and STRIDE (NCT07612878). These studies will further clarify long-term effectiveness, safety and generalisability beyond the pivotal trial populations.

**TABLE 1:** Type 2 inflammation phenotype in COPD.

Domain	Characteristic	Clinical relevance
Biomarkers	Blood eosinophils ≥300 cells/µL (consider ≥150 cells/µL); elevated FeNO; raised total IgE, eotaxin-3, PARC	Identifies patients most likely to benefit from T2-targeted biologics
Clinical features	Chronic bronchitis; frequent moderate/severe exacerbations; corticosteroid responsiveness	Enriches for IL-4/IL-13-driven mucus hypersecretion and airway inflammation
Pathobiology	IL-4/IL-13-mediated eosinophil trafficking, goblet cell metaplasia, mucus hypersecretion, airway remodelling	Explains broad efficacy of dual IL-4/IL-13 blockade beyond eosinophil depletion
Prevalence	20%–40% of COPD patients	Substantial minority warranting systematic screening

COPD, chronic obstructive pulmonary disease; FeNO, fractional exhaled nitric oxide; IgE, immunoglobulin E; PARC, pulmonary and activation-regulated chemokine; T2, type 2. Thresholds shown reflect those used in pivotal biologic trials and are not intended as universal diagnostic criteria for routine care. The 20%–40% prevalence estimate is derived from observational cohorts and may vary by population and definition.

## Dupilumab patient selection

Patient selection for dupilumab in COPD should follow the inclusion criteria validated in the BOREAS and NOTUS trials, adapted to real-world clinical judgement. The pivotal trials enrolled adults aged 40–85 years with a post-bronchodilator FEV_1_/FVC ratio <0.70, FEV_1_ 30%–70% predicted, and a smoking history of at least 10 pack-years. All participants were receiving stable triple inhaled therapy (inhaled corticosteroid/long-acting β_2_-agonist/long-acting muscarinic antagonist) and had evidence of T2 inflammation defined by a blood eosinophil count ≥300 cells/µL at screening (Bhatt et al., 2023; Bhatt et al., 2024; Choi, 2026). Patients were required to have a frequent exacerbator phenotype, defined as at least two moderate or one severe exacerbation in the preceding 12 months, with at least one event occurring despite triple therapy. Chronic bronchitis symptoms, chronic productive cough for at least 3 months, were also required, enriching the population for mucus hypersecretion features plausibly linked to IL-13-driven goblet cell metaplasia (Bhatt et al., 2023; Bhatt et al., 2024; Choi, 2026). Critically, both trials excluded patients with a history of asthma, asthma-COPD overlap, or other pulmonary diseases associated with elevated eosinophils, ensuring that the observed benefits were attributable to COPD itself rather than to misdiagnosed asthma (Bhatt et al., 2023; Bhatt et al., 2024; Choi, 2026).

Table 2 therefore presents the selection criteria in three clearly delineated layers (Oh et al., 2026): criteria supported by the pivotal trials that form the current evidence base for regulatory approval and guideline recommendations (Bhatt et al., 2023); potential real-world expansions that may be considered with clinical judgement; and (Rabe et al., 2023) unresolved evidence gaps that still require prospective validation. In clinical practice, the most suitable candidates are therefore patients with confirmed COPD, persistent exacerbations despite optimized triple therapy, blood eosinophils ≥300 cells/µL, and clinical features of chronic bronchitis. Patients with blood eosinophils between 150 and 299 cells/µL may also be considered if additional T2 features, such as elevated FeNO or a strong corticosteroid response, are present, although prospective validation of this lower threshold is lacking (Christenson et al., 2025b). Conversely, patients with a confirmed diagnosis of asthma or asthma-COPD overlap should be managed according to asthma biologic pathways rather than COPD-specific algorithms, and alternative causes of eosinophilia should be excluded before initiating therapy (Choi, 2026; Matera and Cazzola, 2024). This layered approach underscores that trial eligibility does not automatically equate to a universally validated clinical phenotype for routine prescribing.

**TABLE 2:** Dupilumab patient selection in T2-High COPD: distinguishing trial-supported criteria, real-world considerations and evidence gaps.

Domain	Trial-supported criteria (BOREAS/NOTUS)	Potential real-world considerations	Unresolved evidence gaps
Confirmed COPD diagnosis	Post-bronchodilator FEV_1_/FVC <0.70; FEV_1_ 30%–70% predicted	Same; confirm absence of reversible asthma features	Applicability to milder or more severe airflow limitation
Age and smoking history	40–85 years; ≥10 pack-years	May extend to never-smokers or younger/older patients with clear T2 features in selected cases	Safety and efficacy outside trial age/smoking range
Background therapy	Stable triple inhaled therapy (ICS/LABA/LAMA)	Ensure optimisation; dual therapy acceptable if ICS contraindicated	Optimal sequencing with other add-on therapies
Exacerbation history	≥2 moderate or ≥1 severe in prior 12 months despite triple therapy	Same; focus on frequent exacerbators	Benefit in less frequent exacerbators
Blood eosinophils	≥300 cells/µL at screening	150–299 cells/µL may be considered if elevated FeNO or strong corticosteroid response present	Prospective validation of lower threshold required
Clinical phenotype	Chronic bronchitis (productive cough ≥3 months)	Same; prioritise mucus-hypersecretion features	Benefit in emphysema-predominant phenotypes without chronic bronchitis
Key exclusions	Asthma, asthma–COPD overlap, other eosinophilic lung diseases	Strict adherence recommended; alternative causes of eosinophilia must be excluded	Management algorithms for overlap syndromes

Trial-supported criteria are those validated in the BOREAS, and NOTUS, phase 3 trials and form the evidence base for current regulatory approvals and guideline recommendations. Real-world considerations represent pragmatic extensions that may be applied with clinical judgement but lack prospective validation. Evidence gaps highlight areas where further studies are needed before routine adoption. This table is intended to guide patient selection, not to serve as a validated prescribing algorithm.

## Biomarker stratification and subgroup considerations

Blood eosinophil count remains the primary biomarker for dupilumab patient selection, but post-hoc analyses from the pivotal trials indicate that efficacy extends across eosinophil thresholds and may be augmented by FeNO. A pooled BOREAS/NOTUS analysis showed exacerbation reductions of up to 36.9%, with slightly greater effects at blood eosinophil counts ≥300 cells/µL but significant benefit also observed at ≥150 cells/µL(25). In the BOREAS biomarker analysis, higher baseline eosinophils (P = 0.0056) and higher FeNO (P = 0.043) independently predicted greater treatment response, supporting dual-biomarker strategies for patient selection (Christenson et al., 2025a). Dupilumab also reduced type 2 biomarkers including total IgE (−22.5%), FeNO (−28.6%), eotaxin-3 (−8.8%), and PARC (−14.4%) at week 52, providing direct evidence of pathway modulation (Christenson et al., 2025a). Efficacy was consistent irrespective of BODE index score (Vogelmeier et al., 2025), emphysema presence (Bhatt et al., 2025b), and cough or sputum burden (Hanania et al., 2025), suggesting broad applicability within the T2-high population. An indirect comparison suggested potential advantages for dupilumab over mepolizumab in quality of life, symptoms, and lung function, particularly in patients with FeNO ≥20 ppb; however, the authors of that analysis explicitly noted that the observed differences were statistically but not clinically meaningful and called for direct comparative trials (Suter et al., 2025). These observations imply that while blood eosinophils remain the cornerstone of patient selection, FeNO may add predictive value, particularly in patients with borderline eosinophil counts. However, prospective validation of FeNO-based selection criteria is still required before routine clinical adoption (Christenson et al., 2025b; Xie et al., 2025).

Beyond their statistical association with treatment response, these biomarkers provide mechanistic insight into the biological activity of IL-4 and IL-13 in the COPD airway. Fractional exhaled nitric oxide (FeNO) is produced by airway epithelial cells under the transcriptional control of IL-4 and IL-13 via the Janus kinase–signal transducer and activator of transcription (JAK–STAT) pathway and serves as a real-time, non-invasive readout of airway T2 inflammation (Escamilla-Gil et al., 2022). In COPD, elevated FeNO correlates with greater bronchodilator reversibility and a more favourable response to inhaled corticosteroids, though its levels are generally lower than in asthma and are influenced by smoking, which suppresses FeNO production (Rabe et al., 2023). Total IgE, though non-specific, reflects IL-4-driven B-cell class-switching; its elevation in COPD may indicate atopic tendency or T2 activation, but it is less reliable than eosinophils due to confounding by age, smoking, and concurrent infections (Christenson et al., 2025a). Eotaxin-3 (CCL26) is an IL-13-inducible chemokine that selectively recruits eosinophils to the airway via CCR3; its reduction following dupilumab treatment provides direct evidence that IL-13-driven chemotaxis is being interrupted (Pesic et al., 2025). PARC (CCL18) is produced by alternatively activated macrophages under IL-4/IL-13 stimulation and has been linked to fibrotic and remodelling processes in the lung; its suppression by dupilumab suggests that the biologic may modify structural airway changes beyond acute inflammation. Collectively, these biomarkers are not interchangeable: blood eosinophils capture the effector cell pool, FeNO reflects epithelial signalling, eotaxin-3 reports on chemokine-driven recruitment, and PARC indicates macrophage-derived remodelling signals. For clinical practice, the most immediately applicable dual-biomarker strategy combines blood eosinophils and FeNO, as both are widely available and have been prospectively evaluated in post-hoc analyses (Christenson et al., 2025a). For future trial design, changes in eotaxin-3 and PARC may serve as pharmacodynamic biomarkers to confirm target engagement in early-phase studies, though their utility for patient selection requires prospective validation in dedicated trials.

## Real-world evidence and comparative biologics

Real-world experience with dupilumab in COPD remains limited but is emerging. A single-center compassionate-use case series of 12 elderly patients (mean age 73.6 years) with a chronic bronchitis phenotype reported significant reductions in severe exacerbations (annualized rate 0.75 to 0.19 events/patient-year), FEV_1_ gains of +120 mL at 6 months, and marked improvement in mucus-related symptoms (CAT phlegm item change −2.6) (Mari et al., 2026). A prospective study of 23 patients observed a 55% reduction in annualized exacerbation rate and improved COPD Assessment Test scores, though FEV_1_ did not change significantly (Freund et al., 2025). A Chinese real-world study of 34 patients found FEV_1_ gains of 0.20 L and a reduction in FeNO of 8 ppb over 24 weeks (Chen and Shen, 2026). A population-based cohort study of 1,521 dupilumab initiators reported lower all-cause mortality (HR 0.53), fewer emergency department visits (HR 0.78), and reduced exacerbation rates (HR 0.59) compared with LABA-based therapy (Sun et al., 2025). A retrospective TriNetX comparison of 145 dupilumab and 145 mepolizumab users found no significant differences in exacerbations or healthcare utilization, although mepolizumab showed a non-significant trend toward more emergency department visits (Lee et al., 2026).

Despite these encouraging signals, real-world data remain sparse, and a French cohort analysis found that only 21.7% of real-life COPD patients met trial inclusion criteria for any biologic, with the target population limited to 1.3%–8% depending on the agent (Zysman et al., 2024). A systematic review of real-world and patient-reported outcomes similarly noted fewer than 100 patients across all dupilumab case series, underscoring the need for larger, prospective real-world studies—particularly controlled or registry-based designs that can address confounding by indication (Freund et al., 2024). Despite encouraging signals, real-world evidence remains limited by small sample sizes, predominantly single-centre or retrospective designs, lack of concurrent controls, and potential confounding by indication. The strengths and limitations of the available studies are summarised in Table 3. Larger prospective registry-based studies are still needed to confirm generalisability and long-term outcomes.

**TABLE 3:** Strengths and limitations of published real-world studies of dupilumab in COPD.

Study (ref)	Design/N	Key strengths	Key limitations
(Mari et al., 2026)	Single-centre compassionate use, n = 12	Detailed mucus/symptom data; elderly population	Very small N; no control; short follow-up
(Freund et al., 2025)	Prospective single-centre, n = 23	Prospective data collection; CAT scores	Small N; no concurrent control; FEV_1_ unchanged
(Chen and Shen, 2026)	Single-centre, n = 34	Asian population; FeNO data	Retrospective elements; limited duration
(Sun et al., 2025)	Population-based cohort, n = 1,521	Large N; mortality and healthcare utilisation endpoints	Observational; potential residual confounding
(Lee et al., 2026)	TriNetX matched comparison (dupilumab vs. mepolizumab), n = 145 each	Comparative data	Retrospective; matching limitations
Overall (Freund et al., 2024)	Systematic review of case series	Synthesis of available data	<100 patients total across series; high heterogeneity

The studies listed above represent the currently available real-world evidence for dupilumab in COPD, as of September 2026. All are limited by small sample sizes (except the population-based cohort), predominantly single-centre or retrospective designs, absence of concurrent controls in most series, and potential confounding by indication. The systematic review (Freund et al., 2024) confirms that fewer than 100 patients have been reported across all published case series. Larger prospective, registry-based studies are required to establish generalisability and long-term outcomes.

## Limitations and evidence gaps

The evidence base for dupilumab in T2-high COPD is robust but has important limitations. The BOREAS and NOTUS populations were predominantly White (86.9%) and former smokers (70.2%), and were enriched for chronic bronchitis, limiting generalizability to other racial groups, never-smokers, and emphysema-predominant phenotypes (Matera et al., 2025). The reduction in severe exacerbations did not reach statistical significance in the pooled analysis (RR 0.674, P = 0.073) (Bhatt et al., 2025a), and St George’s Respiratory Questionnaire improvements were inconsistent between the two trials (Bhatt et al., 2023; Bhatt et al., 2024). Blood eosinophil counts correlate poorly with lung tissue eosinophils (Staples et al., 2025), and eosinophil levels can vary over time, complicating threshold-based selection (Criner et al., 2026). Notably, transient blood eosinophilia—a recognized dupilumab effect in asthma—appears absent in COPD, which may limit the utility of post-treatment eosinophil monitoring (Portacci et al., 2025). Educational surveys indicate that pulmonologists remain uncertain about eosinophil thresholds, asthma-COPD overlap, and practical implementation despite guideline endorsements (Boyd et al., 2026).

The most striking gap is the near-absence of controlled real-world data. Fewer than 100 patients are reported across all published dupilumab case series, and no large prospective controlled or registry-based real-world trial has been completed to date, although small single-centre observational studies have begun to appear (Freund et al., 2024). Moreover, the finding that only 1.3%–8% of real-life COPD patients meet trial eligibility criteria raises questions about generalizability that current evidence cannot fully address (Zysman et al., 2024). Biomarker research beyond blood eosinophils—including FeNO, eotaxin-3, and PARC—shows promise but lacks prospective validation (Xie et al., 2025). Long-term safety, durability of response, and effects on mortality remain unresolved, although observational data suggest a possible survival benefit (Sun et al., 2025). Future research should prioritize pragmatic trials in diverse populations, validation of dual-biomarker selection strategies, and longer-term safety surveillance (Ross et al., 2026).

## Future directions

Realizing the full clinical potential of T2-targeted biologics in COPD will require coordinated efforts across several domains.

First, trial design must evolve to address the gap between highly selected trial populations and heterogeneous real-world patients. Pragmatic trials that incorporate broader inclusion criteria—including patients with eosinophil counts down to 150 cells/µL, those with significant emphysema, and those with cardiovascular or metabolic comorbidities—are needed to establish external validity (Zysman et al., 2024). Adaptive platform trials that test multiple biologics against a common control arm could accelerate comparative effectiveness research and reduce the sample size required for each agent.

Second, biomarker development should prioritize the prospective validation of multi-marker strategies. While blood eosinophils and FeNO have emerged as the most promising candidates, their optimal thresholds, dynamic changes over time, and utility in guiding treatment initiation, continuation, or discontinuation remain undefined. Surrogate endpoints, particularly changes in FeNO, eotaxin-3, or PARC, could be used as pharmacodynamic readouts in early-phase trials to confirm target engagement before committing to large outcome trials, but they require formal qualification through regulatory pathways. Lung tissue biomarkers, including sputum eosinophils and bronchial epithelial gene expression signatures, though less accessible, may offer greater specificity and should be incorporated into mechanistic substudies.

Third, unresolved mechanistic questions include the durability of the treatment effect beyond 52 weeks, the effect on lung function decline trajectory (as opposed to acute bronchodilation), and the impact on airway remodelling. The BOREAS and NOTUS open-label extension studies will provide initial data on longer-term outcomes, but dedicated studies with serial imaging and bronchial biopsy are needed to determine whether IL-4/IL-13 blockade can modify structural airway disease. The apparent absence of paradoxical blood eosinophilia, a phenomenon observed with dupilumab in asthma, in COPD warrants further investigation, as it may reflect fundamental differences in the underlying eosinophil biology between the two diseases.

Fourth, health systems and implementation research is critical. The finding that only 1.3%–8% of real-life COPD patients meet trial eligibility criteria does not mean that dupilumab is ineffective in the broader population, but it does indicate that systematic biomarker screening is needed to identify eligible patients. Implementation studies should evaluate the feasibility, cost-effectiveness, and equity of screening programmes in primary, secondary, and tertiary care settings, particularly in low- and middle-income countries where the burden of COPD is highest but access to biologics is most limited.

Finally, head-to-head comparative trials are the logical next step to resolve whether dupilumab offers advantages over mepolizumab or other agents in specific patient subgroups, for example, those with very high FeNO or those with prominent mucus hypersecretion (Sciurba et al., 2025). Such trials should incorporate biomarker-stratified randomisation and prespecified subgroup analyses to identify differential treatment effects that can inform personalised prescribing. Comparative effectiveness among T2-targeted biologics remains an open question. While mepolizumab has also shown benefit in eosinophilic COPD, and agents targeting IL-33 (such as tozorakimab) or other alarmins are in late-stage development, head-to-head trials against dupilumab are still lacking and will be important to define optimal positioning of these therapies.

## Conclusion

The evidence from 2022 onward establishes dupilumab as the first targeted biologic therapy for COPD with type 2 inflammation. Two replicate phase 3 trials, a pooled analysis, and multiple meta-analyses confirm an approximately 30% reduction in moderate or severe exacerbations, clinically meaningful improvements in lung function, and a favourable safety profile in patients selected by blood eosinophils ≥300 cells/µL, frequent exacerbations despite triple therapy, and chronic bronchitis features. Patient selection criteria are well defined but narrow, and real-world evidence remains in its infancy. The convergence of BOREAS, NOTUS, and emerging real-world data positions dupilumab as a paradigm shift toward precision medicine in COPD. Realizing its full clinical potential will require expanded biomarker validation, studies in diverse populations, and pragmatic trials that bridge the gap between trial eligibility and real-world applicability.

## Footnotes

1 Global Initiative for Chronic Obstructive Lung Disease. Global Strategy for the Diagnosis, Management, and Prevention of Chronic Obstructive Pulmonary Disease: 2026 Report. 2026. Available from: https://goldcopd.org/2026-gold-report/
